# Supplementary material for: Association between physical activity levels and functional recovery, quality of life, and psychological well-being in patients undergoing physiotherapy for musculoskeletal disorders: A cross-sectional study
Source: PLoS One. 2026 Feb 9;21(2):e0341335. doi: 10.1371/journal.pone.0341335 (PMC12885262; doi:10.1371/journal.pone.0341335)
Supplement: Attachment — Submitted filename: Response to Reviewers.pdf [file pone.0341335.s003.pdf]

Point-to-point author response to editor and Reviewer Comments

Editorial Assessment:
This manuscript focuses on a clinically relevant and globally important issue - the effect of different levels of physical activity (PA) in the functional recovery and quality of life (QoL) and psychological change of people with musculoskeletal disorders undergoing physiotherapy. The paper is well written, well organized, and provides suitable statistical analyses. It provides methodological rigor in a number of ways, including the use of validated outcome measures (PSFS, SF-36, HADS) and the use of multivariable modeling with moderator analysis.
However, there are a number of substantive issues to consider in terms of clarity of study design, clarity of reporting methodology and transparency of data, statistical validity and consistency across sections. These issues must be deal with before the manuscript can be considered for publication. Some weaknesses are associated with the overinterpretation of cross-sectional results, inconsistent reporting of the results of analyses and unclear coding of variables.
With overtaking, this manuscript is capable of providing a useful contribution.
Major Comments (Require major revision)
Query: 1. Study Design Limitations Are Under Reported
This is a cross-sectional study, but in the manuscript, there is the repeated use of causal or quasi-causal terms (e.g. "physical activity contributed to recovery", improved outcomes", "dose-response relationship. Cross-sectional data is unable to find direction or impact, only association.
Required:
Unique and discriminate to study claims: - “Server Finding”: - Theme of interest: - Major findings: - Claim: Due to the study, it is considered that there are some of the following ideas. Cloud or Version Cloud” Unique to the Studies of Claims: build Reset throughout the Abstract, Results, and Discussion and Conclusion of some version and association, the ideas of claim chain not cause.
Looking more specifically at studies of definitely carcinogenic substances, this would be a relevant consideration: according to Takano, "It has to be made clear that 'dose--response' is observational and is not causal."
Author’s Response: The manuscript has been revised to address the overinterpretation of cross-sectional findings by eliminating causal or quasi-causal language and replacing it with terminology appropriate for observational associations. Phrases such as “contributed to recovery,” “improved outcomes,” and “dose–response relationship” have been amended to reflect associations, trends, or patterns rather than causal implications. The revisions have been applied consistently across the Abstract, Results,

Discussion, and Conclusion sections to ensure alignment with the study's cross-sectional design and its inherent limitations. An explicit statement regarding the non-causal nature of the findings and the observational nature of any detected associations has also been inserted into the Discussion.

Query: 2. Physical Activity Grouping Is Not Described in Sufficient Detail
The manuscript says that the subjects were divided into low, moderate and high activity groups but:
Cutoff METs No MET cutoffs used by the study are provided.
•It is not clear if classification was based on WHO, IPAQ scoring guidelines or custom thresholds.
Some of the non-technical reasons for using the thresholds are lacking (e.g., rationale for thresholds and distribution of participants into groups is missing).
Required:
Include exact MET values (i.e. "Low < X MET-min/week", etc.) and percentages of the participants in each category.
Author's Response: The manuscript has been revised to include the specific MET-min/week cutoffs used to categorize physical activity levels into low, moderate, and high groups, following the official scoring protocol of the International Physical Activity Questionnaire – Short Form (IPAQ-SF). A justification for using these thresholds is now provided, referencing their validation in international and Arabic-speaking populations. Additionally, the percentage distribution of participants across the three physical activity categories has been added to clarify group composition.

Query: 3. Potential Sampling Bias and Recruitment Procedure Should be Made Clear
Purposive sampling occurs in one tertiary treatment hospital may introduce several selection biases.
Concerns:
. No explanation of how many patients were screened vs. enrolled.
.Flow diagram (CONSORT-style suggested for PLOS ONE also for observational studies).
The report does not provide any detailed information on refusal rate or non-response characteristics.
Required:
Add participant flow information, explanation of sampling strategy limitations.
Author's Response: The manuscript has been updated to address the potential for sampling bias by clarifying the recruitment process, including the number of patients screened, enrolled, and excluded. A brief justification of the purposive sampling strategy and its limitations is now included in the *Participants* section. To enhance

transparency, a participant flow diagram consistent with PLOS ONE recommendations for observational studies has also been added as *Figure 1*.

Query: 4. Multiple Variables Have No operational definitions
For example:
Baseline severity" is used in the model and never defined or described.
•"High socioeconomic status" does not have the exact classification criteria.
Medications used: Which medications? For what indications? How measured?
•Comorbidities - grouped as binary, but later described by authors as moderators.
Required:
Provide definitions and procedures for the measurement of these variables.
Author's Response: The manuscript has been revised to include precise operational definitions and measurement procedures for all key variables mentioned in the statistical models. Definitions for *baseline severity*, *socioeconomic status*, *medication use*, and *comorbidities* have been added to the **Variables** section. This ensures clarity regarding how each variable was assessed, categorized, and used in the analysis, particularly in regression and moderation models.

Query: 5. Statistical Analysis Section Overstates Rigor
Issues include:
o Claims regarding the multicollinearity checks without reporting on values of VIF. Hypothesis Testing in Logistic Regression Model Fitting 1: Empirical Analysis and Interpretation Part III Model Testing 1. Model comparing F Statistics adjusted R2 for model Adjusted R2 per model 2. Residual versus Fitted 3. Effect of Variance 4. Residual vs. Fitted: Adding Polynomial basis & Interaction of two variables 5. Residual vs Fitted 6. Significance vs. Random Effects 7. Effect of Variance Logistic Regression Model Fitting Part III Model Testing 1.
Model parameters are wanting, particularly for exact models whereas interaction terms are described for moderation models.
•Report of incomplete (missing means, SD, post-hoc p-values and confidence intervals) for AN/ANCOVA.
Required:
Provide complete statistical reporting using PLOS ONE and APA guidelines.
Author's Response: The manuscript has been revised to improve the transparency and completeness of statistical reporting in accordance with **PLOS ONE** and **APA** guidelines. Variance inflation factor (VIF) values are now explicitly reported to support the multicollinearity checks. Full model parameters, including **adjusted R^2**, **F-statistics**, **confidence intervals**, **effect sizes**, and **post-hoc p-values**, are now provided where applicable for both ANOVA and ANCOVA results. Details of the moderation models

have also been clarified by listing specific interaction terms and their statistical contributions. These additions ensure statistical validity and improve interpretability.

Query: 6. Figures and Tables Incomplete Correspondence with the Narrative
Examples:
Table 1 reports on many of the variables that are not included in the main regression.
Of the above variables, the following are listed in Table 2 as not elaborated on as a priori model in methods:
Figures are mentioned in the text but not clearly described and/or labelled in the manuscript
Required:
Ensure consistency between:
Methods-> Statistical Analysis -> Tables-> Results.
Author's Response: The manuscript has been revised to ensure complete alignment and consistency between the **Methods**, **Statistical Analysis**, **Results**, **Figures**, and **Tables**. All variables included in regression and moderation models are now clearly specified in the *Statistical Analysis* subsection as a priori covariates or moderators. Variables listed in **Table 1** but not used in the main models (e.g., smoking, employment status) have been addressed explicitly to distinguish descriptive reporting from inferential analysis. Figure captions have been expanded for clarity, and in-text references to figures and tables have been revised to match their order and content precisely.

Query: 7. Overreliance on Self Report Measures
The study acknowledges this limitation, but there is no discussion of its full implications, in particular:
•Social desirability bias
Overprediction of MET min in IPAQ-SF
•Recall bias
Methods: - Possibility that poor psychological status decreases reporting of physical activity
Required:
Strengthen the limitations section and its meaning for interpretation;
Author's Response: The *Limitations* section has been expanded to address the broader implications of using self-report measures, particularly regarding the potential impact of social desirability bias, recall bias, and psychological state on the accuracy of reported physical activity and related outcomes. These limitations are now contextualized in terms of their influence on data interpretation, especially the possibility of overestimation of physical activity and underreporting of psychological symptoms in certain subgroups.

Query: 8. Citations Need Reviewing for Accuracy and Relevance
Several citations are not used to support the claims in the text. Examples:
Some references refer to surgical recovery, frailty or a cancer population - less relevant about physiotherapy to musculoskeletal disorders.
•Some are seemingly out of step with statements (e.g. a citation on sodium-glucose inhibitor prescriptions to support SES effects).
Required:
Review and correct references to help ensure they are supporting the particular statements.
Moderate/Structural Issues
Author's Response: All citations have been systematically reviewed for accuracy, relevance, and contextual fit. References that pertained to surgical, cancer, or pharmacological populations not directly related to musculoskeletal physiotherapy have been removed or replaced with more appropriate literature. Specifically, citations previously used to support claims regarding the influence of socioeconomic status, physical activity effects, or recovery outcomes have been revised to ensure they accurately reflect the target population and constructs discussed in the manuscript. Citation alignment across the *Introduction*, *Discussion*, and *References* sections has been updated accordingly.

Query: 9. The Introduction is Too Long and Repetitive
Several paragraphs repeat similar points of background information about the benefits of physical activity.
Suggestion: Fewer to sentence - 3-4 short, connected paragraphs.
Author's Response: The *Introduction* has been revised to eliminate redundancy and improve focus. Repetitive statements about the general benefits of physical activity have been condensed, and the structure has been streamlined into four concise, thematically connected paragraphs. The revised version maintains the necessary background while providing a clear rationale for the study and its research questions without reiteration.

Query: 10. Results Section Needs to be Clearer
PLOS ONE is fairly lenient when it comes to p values lacking effect sizes.
No indication whether tests for assumptions of linear regression (normality, homoscedasticity) were calculated.
Required: Input for assumption checks, important effect size measures
Author's Response: The *Results* section and *Statistical Analysis* subsection have been updated to report diagnostic checks for linear regression assumptions, including normality, linearity, homoscedasticity, and absence of multicollinearity. All models

were tested using residual plots and statistical diagnostics, with no violations observed. Additionally, effect sizes (standardized beta coefficients and partial eta squared) have been clearly reported alongside p-values to enhance interpretability in accordance with PLOS ONE and APA standards.

Query: 11. Wording Throughout Suggests Generalization Across Sample
Claims which seem to generalize findings to more general clinical populations should be moderated.
Author's Response: The manuscript has been revised to moderate language that may have implied unwarranted generalization beyond the study sample. Phrasing throughout the *Abstract*, *Discussion*, and *Conclusion* sections has been adjusted to reflect the specificity of the findings to the study's cross-sectional design, setting, and sample characteristics. Terms such as "suggests," "may be associated," and "in this sample" have been used to ensure claims remain grounded in the observed data without overextending to broader clinical populations.

Query: 12. Ethical Approval Statement Should Make Clear Prospective or Retrospective Recruitment
This is required in PLOS ONE.
Author's Response: The ethical approval statement has been updated to clarify that participant recruitment and data collection were conducted **prospectively** following Institutional Review Board (IRB) approval. This revision aligns with PLOS ONE's reporting requirements and clearly communicates the temporal sequence of ethical clearance and recruitment.

Minor Issues
Query: 13. Minor Language Issues
The manuscript is generally well written but includes:
•Repetition ("well-documented" is repeated too often)
Excessive use of the phrase "these findings highlight"
•Occasional grammar problems (e.g. article, inconsistent tense)
A polish pass of the language is recommended.
Author's Response: A thorough language revision has been completed to improve clarity, eliminate redundancy, and correct minor grammatical inconsistencies. Repetitive phrases such as "well-documented" and "these findings highlight" have been replaced with varied, context-appropriate alternatives. Article usage, verb tense consistency, and sentence structure have been reviewed throughout the manuscript to ensure grammatical accuracy and stylistic refinement.

Query: 14. Formatting Issues
Example: For example, "Click here to access/download" should be replaced with nicer text.
Documentary elements must adhere to the structure of PLOS ONE for: - Caption to figure.
Include the following in your answer in the rubric: "please show consistency in reporting decimals" 2 vs. 3 decimal places
Author's Response: Formatting throughout the manuscript has been revised to meet PLOS ONE submission standards. Phrases such as "Click here to access/download" have been removed or replaced with appropriate descriptive text. Figure captions have been standardized to provide complete, standalone descriptions in accordance with journal guidelines. Additionally, all numeric values have been reviewed to ensure consistent decimal reporting, with all statistical values (e.g., means, SDs, p-values, confidence intervals) now presented uniformly to two decimal places, unless three are necessary for clarity (e.g., $p < 0.001$).

Query: 15. Data Availability
For this case, the DOI for Zenodo is provided. Confirm the completeness of data sets as well as clarity of codebook
Author's Response: The Zenodo repository linked in the manuscript has been reviewed to ensure full compliance with data availability standards. The dataset is complete and includes all variables analyzed in the manuscript. A clearly labeled, structured codebook is provided as a separate file within the repository, detailing variable names, descriptions, coding schemes (e.g., binary and categorical values), and measurement units. These elements collectively ensure transparency and enable reproducibility of the reported analyses.

Recommendation
Decision: MAJOR REVISION
The manuscript discusses an important topic and presents appropriate standardized tools; however, significant revision is necessary to make certain:
Appropriate interpretation of cross-sectional data
Transparency in reporting method and statistical procedures
Definitive explanations of variables
Improved coherence between sections
Limited and discussed biases
If the above points are taken seriously by the authors, the manuscript will certainly be much stronger and will be suitable for publication.

Author's Response: All requested areas for revision have been addressed to ensure the manuscript meets the expectations outlined. Specifically:

- Interpretation of Cross-Sectional Data: All causal or quasi-causal language has been replaced with terminology reflecting correlational relationships. Statements have been revised to clarify that findings are associative and context-specific, not generalizable or indicative of causality. See revisions in the *Abstract*, *Results*, *Discussion*, and *Conclusion* sections.
- Transparency in Methods and Statistical Reporting: Assumptions for regression models (normality, linearity, homoscedasticity, multicollinearity) have been explicitly tested and reported. Effect sizes, model parameters, F-statistics, confidence intervals, and post hoc comparisons are now included in tables and narrative summaries. VIF values have been reported to support multicollinearity checks (see *Statistical Analysis* and *Results*).
- Operational Definitions of Variables: Key variables such as baseline severity, socioeconomic status, comorbidities, and medication use have been clearly defined in the *Variables* section, including how each was measured, coded, and used in analyses.
- Coherence Across Sections: The manuscript structure has been refined to ensure alignment between the *Methods*, *Results*, *Figures*, and *Tables*. Inconsistencies in the inclusion and interpretation of variables have been resolved. Citations were reviewed and revised to ensure they accurately support the associated claims.
- Bias and Limitations: The *Limitations* section has been expanded to reflect potential biases related to self-reporting, including recall bias, social desirability bias, and misclassification. The impact of these limitations on the interpretation of findings is now explicitly discussed.

With these comprehensive revisions implemented, the manuscript now presents a coherent, transparent, and methodologically sound account of the research, suitable for publication pending final editorial approval.

Point to-point Responses yto reviewer queries (Dr. Paiwand Mamand Sultan)

Query: The manuscript is well-written, uses validated outcome tools (IPAQ-SF, PSFS, SF-36, HADS), includes ethical approval, a data availability statement, and a clear statistical approach.
However, major methodological and reporting issues must be addressed before acceptance.
My recommendation: **MAJOR REVISION**
Author's Response: All methodological and reporting issues noted will be fully addressed in the revised manuscript. We have ensured that validated instruments are used appropriately, ethical standards are adhered to, and statistical methods are clearly reported. Specific clarifications, corrections, and additions have been made as outlined in the responses to each individual comment below.

Query: Abstract
The abstract is scientifically correct, but it is too long and contains excessive statistical details, as the abstract should focus on the findings only. And a lack of essential clarity regarding PA categorization and group sizes. Also, keywords are fine but could be reduced or prioritized.
Author's Response: The abstract has been revised to improve clarity and conciseness by reducing statistical detail and emphasizing key findings. Physical activity (PA) categorization and group sizes have been clearly specified. Keywords have been refined to include only the most essential terms.

Query: Introduction
The introduction is repetitive and could be more concise; several concepts, such as the importance of physical activity, its role in chronic disease, and its association with MSK outcomes, appear multiple times.
The rationale is too general and does not clearly identify a specific research gap. It does not specify which populations are understudied. It does not clarify which outcomes (function, QoL, Psychological) lack evidence.
Some background citations are not closely aligned with the study question. Such that reference to schoolyard PA measurement (Ref 10), COVID-19 psychological outcomes (Ref 29, 30), Cardiac surgery PA recovery factors (Ref 31).
The introduction overemphasizes general PA benefits rather than MSK-specific evidence. It focuses heavily on global physical activity recommendations and broad public health arguments, rather than on mechanistic links between PA and MSK recovery, recovery

trajectories in MSK physiotherapy, or prior MSK rehabilitation studies assessing PA, QoL, or psychological factors.

The aim is clearly stated, but the hypotheses are not aligned with the cross-sectional design. The introduction suggests PA "improves" or "enhances" recovery, which implies causation. This is not appropriate for cross-sectional studies, which only examine associations.

Author's Response: The introduction has been revised for conciseness and clarity in response to both this and a prior reviewer's comment. Repetitive content was removed, and general references to public health and non-MSK-related studies have been excluded. The revised version focuses specifically on musculoskeletal (MSK) rehabilitation, clearly identifies the research gap regarding variable physical activity levels and their associations with functional, quality of life, and psychological outcomes in this population. The aim remains clearly stated, and the hypothesis wording has been adjusted to reflect the cross-sectional design by avoiding causal language.

Query: Methods

The section clearly reports the study setting, IRB approval, informed consent, and compliance with ethical standards. However, additional details are needed regarding the clinical environment, recruitment procedures, and confidentiality. The long data collection period should be justified, especially given the cross-sectional nature of the study.

The manuscript claims: Cross-sectional study correct, but the Methods contain procedures that are not cross-sectional, e.g., "Participants… who could commit to follow-up assessments" (L120-126), Weekly activity logs to "reduce recall bias" (L171-174).

Physical activity measurement is unclear and inconsistent. You state: Physical activity is calculated in MET hours/week, but IPAQ is normally scored in MET-min/week, not hours. This creates confusion: Did authors convert MET-min to MET-hours? Was the IPAQ scoring protocol strictly followed?

Regression model overfitting and multicollinearity concerns, this model includes too many predictors relative to sample size: Age, Gender, Socioeconomic status, Comorbidities, Baseline health status, Medication use, BMI, Employment, Smoking, Physical activity

Although they checked VIF, adding many interaction terms with only 286 participants may produce unstable estimates, so it needs justification for the number of predictors or performing stepwise.

Also, outcome variable definitions lack detail. For PSFS: you changed the scaling ("higher score = better function"). But PSFS traditionally takes the mean of 3–5 activities selected by patients. Did everyone choose the same number of activities? How many activities were used? How was the final score calculated? Missing details reduce reproducibility.

In Methods, remove overly detailed narrative text. E.g., long descriptions about the training of research assistants.

Author's Response: The Methods section has been revised to clarify the clinical environment, recruitment procedures, and participant confidentiality. The data collection period is now justified in light of clinical flow and recruitment pace. References to "follow-up assessments" and weekly logs have been removed or rephrased to avoid any implication of longitudinal design. Physical activity measurement has been clarified, specifying the conversion from MET-min/week to MET-hours/week and adherence to IPAQ scoring protocols. The Patient-Specific Functional Scale (PSFS) scoring method is now explicitly described, including the number of activities selected and calculation of final scores. To address regression model complexity, justification for the number of predictors has been added, and variance inflation factor (VIF) checks are retained to support model stability. Finally, excessive procedural detail, such as training of research assistants, has been removed for conciseness.

Query: Results

Statistical inconsistencies. Several inconsistencies were noted:

- R-values and p-values do not always match typical effect size patterns.
- Table 3 shows SD next to R-values, which is unusual and unclear.
- Regression coefficients inconsistent between text and tables (e.g., β=0.36 vs β=0.38).

These should be double-checked with the raw dataset.

Also, the figures are too simple and do not show the required element as expected:

- Confidence intervals
- Detailed moderation plots
- Clarification of axes and units

Figures provided are basic and need improvement.

Tables need clearer labelling. E.g., Table 3 should not display SD for correlation analysis.

Author's Response: All statistical values have been cross-checked with the raw dataset to resolve inconsistencies. Regression coefficients and p-values have been corrected for consistency between text and tables. In Table 3, standard deviations (SD) have been removed from the Pearson correlation outputs, as they are not appropriate for this analysis. Figure captions and figure content have been revised to include confidence intervals, labeled axes (including units), and properly formatted moderation plots with interaction lines. All tables have been relabeled for clarity and adherence to reporting standards.

Query: Clinical characteristics lack specificity

The diagnosis categories (lower back pain, OA, injuries) are too broad. For example: What proportion had each diagnosis? Were severity scores included (e.g., NRS pain)? "Baseline health status" is used as a regression variable, but NOT defined.

Author's Response: The manuscript has been revised to specify the proportions of major diagnostic categories within the study cohort. Additionally, the term "baseline health status" has been defined and clarified in the Methods section. Severity scores, including the Numeric Rating Scale (NRS) for pain, were not collected and are now acknowledged as a limitation in the Discussion.

Query: Ethical Approval

Ethical approval is clearly stated. Data is available in Zenodo. Author contributions and funding are complete. But STROBE guidelines need stronger adherence. Specifically: Participant flow diagram missing, missing description of handling potential biases, and missing justification for the missing data handling approach.

Author's Response: To strengthen adherence to STROBE guidelines, the manuscript now includes: (1) a participant flow diagram outlining eligibility screening, exclusions, and final inclusion; (2) a description of potential sources of bias and the methods used to minimize them; and (3) a justification for the handling of missing data, which involved listwise deletion due to low overall missingness (<5%). These additions are incorporated in the Methods section, and the new figure has been added as Supplementary Figure 1.

Query: References

Some references are outdated (older than 5 years). Although PLOS ONE accepts any year, the research justification should rely more on recent studies.

Author's Response: The introduction and background sections have been revised to replace or supplement older references with more recent and clinically relevant literature (published within the last five years) to strengthen the scientific rationale. This includes updated evidence regarding physical activity and its association with musculoskeletal rehabilitation outcomes, psychological well-being, and recovery trajectories. Outdated references not central to the argument have been removed.
