# Supplementary material for: Association between physical activity levels and functional recovery, quality of life, and psychological well-being in patients undergoing physiotherapy for musculoskeletal disorders: A cross-sectional study
Source: PLoS One. 2026 Feb 9;21(2):e0341335. doi: 10.1371/journal.pone.0341335 (PMC12885262; doi:10.1371/journal.pone.0341335)
Supplement: Attachment — Submitted filename: PLOS one review points.docx [file pone.0341335.s001.docx]

The suggestions for the paper: **Association Between Physical Activity Levels and Functional Recovery, Quality of Life, and Psychological Well-Being in Patients Undergoing Physiotherapy for Musculoskeletal Disorders: A Cross-Sectional Study.**

The manuscript is well-written, uses validated outcome tools (IPAQ-SF, PSFS, SF-36, HADS), includes ethical approval, a data availability statement, and a clear statistical approach.

However, major methodological and reporting issues must be addressed before acceptance.

My recommendation: **MAJOR** **REVISION**

1. **Abstract**

The abstract is scientifically correct, but it is too long and contains excessive statistical details, as the abstract should focus on the findings only. And a lack of essential clarity regarding PA categorization and group sizes. Also, keywords are fine but could be reduced or prioritized.

2. **Introduction**

The introduction is repetitive and could be more concise; several concepts, such as the importance of physical activity, its role in chronic disease, and its association with MSK outcomes, appear multiple times.

The rationale is too general and does not clearly identify a specific research gap. It does not specify which populations are understudied. It does not clarify which outcomes (function, QoL, Psychological) lack evidence.

Some background citations are not closely aligned with the study question. Such that reference to schoolyard PA measurement (Ref 10), COVID-19 psychological outcomes (Ref 29, 30), Cardiac surgery PA recovery factors (Ref 31).

The introduction overemphasizes general PA benefits rather than MSK-specific evidence. It focuses heavily on global physical activity recommendations and broad public health arguments, rather than on mechanistic links between PA and MSK recovery, recovery trajectories in MSK physiotherapy, or prior MSK rehabilitation studies assessing PA, QoL, or psychological factors.

The aim is clearly stated, but the hypotheses are not aligned with the cross-sectional design. The introduction suggests PA “improves” or “enhances” recovery, which implies causation. This is not appropriate for cross-sectional studies, which only examine associations.

3. **Methods**

The section clearly reports the study setting, IRB approval, informed consent, and compliance with ethical standards. However, additional details are needed regarding the clinical environment, recruitment procedures, and confidentiality. The long data collection period should be justified, especially given the cross-sectional nature of the study.

The manuscript claims: Cross-sectional study correct, but the Methods contain procedures that are not cross-sectional, e.g., “Participants… who could commit to follow-up assessments” (L120-126), Weekly activity logs to “reduce recall bias” (L171-174).

Physical activity measurement is unclear and inconsistent. You state: Physical activity is calculated in MET hours/week, but IPAQ is normally scored in MET-min/week, not hours. This creates confusion: Did authors convert MET-min to MET-hours? Was the IPAQ scoring protocol strictly followed?

Regression model overfitting and multicollinearity concerns, this model includes too many predictors relative to sample size: Age, Gender, Socioeconomic status, Comorbidities, Baseline health status, Medication use, BMI, Employment, Smoking, Physical activity

Although they checked VIF, adding many interaction terms with only 286 participants may produce unstable estimates, so it needs justification for the number of predictors or performing stepwise.

Also, outcome variable definitions lack detail. For PSFS: you changed the scaling (“higher score = better function”). But PSFS traditionally takes the mean of 3–5 activities selected by patients. Did everyone choose the same number of activities? How many activities were used? How was the final score calculated? Missing details reduce reproducibility.

In Methods, remove overly detailed narrative text. E.g., long descriptions about the training of research assistants.

4. **Results**

Statistical inconsistencies. Several inconsistencies were noted:

- R-values and p-values do not always match typical effect size patterns.
- Table 3 shows SD next to R-values, which is unusual and unclear.
- Regression coefficients inconsistent between text and tables (e.g., β=0.36 vs β=0.38).

These should be double-checked with the raw dataset.

Also, the figures are too simple and do not show the required element as expected:

- Confidence intervals
- Detailed moderation plots
- Clarification of axes and units

Figures provided are basic and need improvement.

Tables need clearer labelling. E.g., Table 3 should not display SD for correlation analysis

5. **Clinical characteristics lack specificity**

The diagnosis categories (lower back pain, OA, injuries) are too broad. For example: What proportion had each diagnosis? Were severity scores included (e.g., NRS pain)? "Baseline health status” is used as a regression variable, but NOT defined.

6. **Ethical Approval**

Ethical approval is clearly stated. Data is available in Zenodo. Author contributions and funding are complete. But STROBE guidelines need stronger adherence. Specifically: Participant flow diagram missing, missing description of handling potential biases, and missing justification for the missing data handling approach.

7. **References**

Some references are outdated (older than 5 years). Although PLOS ONE accepts any year, the research justification should rely more on recent studies.

Thank You.

Paiwand Mamand Sultan

MSc of Musculoskeletal Physiotherapy.
